# A new diplommatinid genus and two new species from the Philippines (Gastropoda, Caenogastropoda, Cyclophoroidea)

**DOI:** 10.3897/zookeys.678.13059

**Published:** 2017-06-06

**Authors:** Barna Páll-Gergely, András Hunyadi, Takahiro Asami

**Affiliations:** 1 Department of Biology, Shinshu University, Matsumoto 390-8621, Japan; 2 Adria sétány 10G 2/5., Budapest 1148, Hungary

**Keywords:** Land snail, Luzon, rock habitat, systematics, taxonomy

## Abstract

A new diplommatinid genus, *Luzonocoptis* Páll-Gergely & Hunyadi, **gen. n.** is erected for two new species, *Luzonocoptis antenna* Páll-Gergely & Hunyadi, **sp. n.** and *L. angulata* Páll-Gergely & Hunyadi, **sp. n.** Both species inhabit the northeastern part of Luzon Island, Philippines. The genus *Luzonocoptis* **gen. n.** is mostly characterized by a very slender shell with 14–18 whorls, a strongly expanded peristome, an interrupted, weak columellar lamella, the absence of any additional plicae or lamellae, and a rachidian tooth having five cusps.

## Introduction

Diplommatinidae (Caenogastropoda: Cyclophoroidea) are mostly characterized by small shells, a body whorl with a distinctive constriction, and teeth or plicae, which are situated inside the last whorl. This family is widely distributed in eastern and southern Asia, northern Australia, the Pacific islands, and some isolated distributions in South America and Madagascar (Kobelt 1902, Thiele 1929, Wenz 1939, Haas 1961, Simone 2013, Egorov 2013).

The Diplommatinidae of the Philippines were mainly described in 12 papers by Otto von Möllendorff (von Möllendorff 1887a, 1887b, 1887c, 1887d, 1888a, 1888b, 1890a, 1890b, 1891, 1893, 1898, Quadras and von Möllendorff 1893–1896). Although many species were described without figures, Zilch (1953) published photos of all known Philippine diplommatinids. For more than a century, since the last description, no information on the Philippine members of this family has been published. Recently, Poppe et al. (2015) described two species of the Diplommatinidae from the Philippines.

To date, the following species have been described from Cagayan Province, northern Luzon island: *Palaina conspicua* Möllendorff, 1893, *P. conspicua versicolor* Möllendorff, 1893, *P. cristata* Quadras & Möllendorff, 1893, *Diplommatina cagayanica* Möllendorff, 1893, *D. latilabris* Kobelt, 1886, Diplommatina (Sinica) concolor Quadras & Möllendorff, 1893, and D. (S.) filicostata Möllendorff, 1893) (see Zilch 1953). In this paper, two new rock-dwelling diplommatinid species are described from Cagayan Province, which possess characteristic unusual shell characters, not known in any other genera of the family. Therefore, a new genus, *Luzonocoptis* gen. n. is erected for them.

## Materials and methods

Determination of number of shell whorls (precision to 0.25 whorl) follows Kerney and Cameron (1979: 13). The radulae of two specimens were examined. Individual desiccated bodies were soaked in 2 M KOH solution overnight before extracting the radula, which was preserved in 70 % ethanol. Shells, operculae and radulae were directly observed without coating under a low vacuum SEM (Miniscope TM-1000, Hitachi High-Technologies, Tokyo). Measurements of the shell were taken as follows:

**shell width** diameter of the penultimate whorl perpendicular to coiling axis;

**shell height** length from apical tip to the edge of the basal section of the peristome parallel to coiling axis;

**aperture height** length from upper palatal to basal section of peristome parallel to coiling axis.

The mostly widely used terms were used in the descriptions, with the exception of the following: “post-constriction bay” refers to the widened area just anterior to operculum; “neck region” indicates the part of the body whorl on the opposite (“back”) side of the aperture.

### Abbreviations

**D** shell diameter

**H** shell height

**HA** collection András Hunyadi (Budapest, Hungary)

**HNHM** Hungarian Natural History Museum (Budapest, Hungary)

**PGB** Collection Barna Páll-Gergely (Mosonmagyaróvár, Hungary)

## Systematic part

### Diplommatinidae Pfeiffer, 1856

#### Genus Luzonocoptis

Taxon classificationAnimaliaArchitaenioglossaDiplommatinidae

Páll-Gergely & Hunyadi gen. n.

http://zoobank.org/A1561D65-F5BF-47B1-90B4-D10B9E913CF8

##### Diagnosis.

Shell sinistral; apex blunt, club-like; shell very slender with 14–18 whorls, rather regularly, finely ribbed; aperture round with a weak columellar lamella visible from standard apertural view; columellar lamella interrupted, its inner, short portion blunt thorn or tubercle-like, situated inside post-constriction bay; other inner plicae and lamellae absent; outer surface of operculum matt, smooth; inner surface with a very slightly elevated arcuate ridge; rachidian tooth with five cusps (central one blunt, larger than other four cusps), marginal teeth with four pointed cusps.

##### Differential diagnosis.

*Luzonocoptis* gen. n. differs from *Palaina* Semper, 1865 (type species: *Diplommatina macgillivrayi* Pfeiffer, 1854) by the unique shell shape, the strongly expanded peristome, and most importantly, the presence of a columellar tooth, which continues to a strongly developed lamella (see Yamazaki et al. 2013 and Neubert and Bouchet 2013). The most similar diplommatinid genus in terms of shell characters is *Hungerfordia*. *Luzonocoptis* gen. n. differs from *Hungerfordia* by the presence of an interrupted columellar lamella, and the rachidian tooth, which possess five well-developed cusps. In contrast, the columellar lamella of *Hungerfordia* is not interrupted, and the rachidian tooth is simpler, with a single, or three cusps.

##### Etymology.

The first part of the name derives from the name of the island (Luzon), where the included new species have been found. The second part (“-coptis”) refers to the similarity with Middle American urocoptid taxa in terms of shell size, shape, colour and habitat. Gender feminine.

##### Type species.

*Luzonocoptis antenna* sp. n.

##### Content.

*Luzonocoptis antenna* sp. n. and *L. angulata* sp. n.

##### Distribution.

This genus is known so far from northeastern Luzon Island. The distance between the type localities of the two species is approximately 34 km in a straight line.

#### Luzonocoptis antenna

Taxon classificationAnimaliaArchitaenioglossaDiplommatinidae

Páll-Gergely & Hunyadi sp. n.

http://zoobank.org/842D519D-51D9-496F-9422-5806F625E234

**Figure 1.**
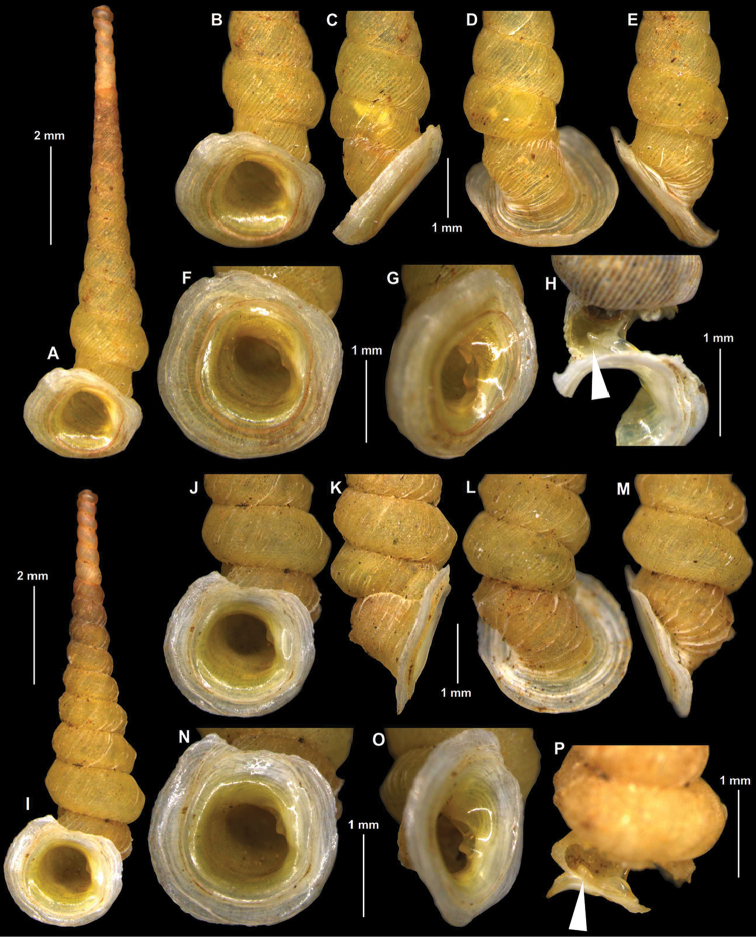
Shells of *Luzonocoptis* gen. n. species. **A–H** *Luzonocoptis antenna* sp. n. (**A–G** holotype HNHM 99995 **H** paratype HNHM 99997) **I–P** *Luzonocoptis angulata* sp. n. (**I–O** holotype HNHM 99996 **P** paratype HNHM 99998). Arrows indicate the inner, separate portion of the columellar lamella. All photos B. Páll-Gergely.

**Figure 2.**
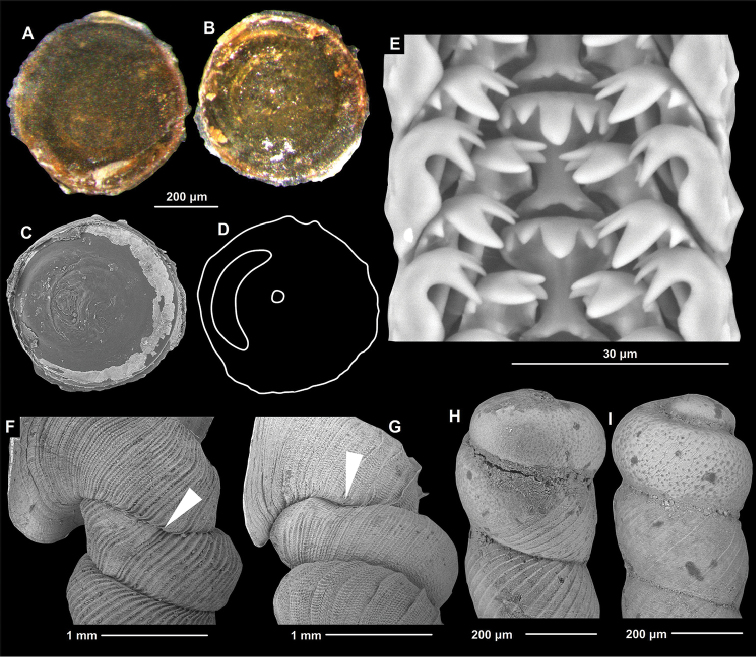
Shells, operculum and radula of *Luzonocoptis* gen. n. species. **A–F** *Luzonocoptis antenna* sp. n. **A** outer side of the operculum **B** inner surface of the operculum **C** SEM of the inner surface of the operculum **D** schematic drawing of the inner side, showing the slightly elevated ridge and the central nipple **E** radula (paratype no. 7.) **F** Neck region **G** Neck region of *Luzonocoptis angulata* sp. n. **H** protoconch of *Luzonocoptis antenna* sp. n. **I** protoconch of *Luzonocoptis angulata* sp. n. White arrow shows the constriction. All images B. Páll-Gergely.

Figures 1A–H , 2A–F, H

##### Type material.

Philippines, Luzon, Cagayan Province, 20 km south-southeast from Baggao, Barangay San Miguel, environment of the Duba Cave, limestone rock wall on the bank of the Pared River, 50 m, 17°49.967'N, 121°56.042'E, leg. Hunyadi, A., 07.01.2014., HNHM 99995 (holotype, H = 9.4 mm, D = 1.7 mm), HNHM 99997 (5 paratypes), HA/166 paratypes, PGB/3 paratypes.

##### Type locality.

Philippines, Luzon, Cagayan Province, 20 km south-southeast from Baggao, Barangay San Miguel, environment of the Duba Cave, limestone rock wall on the bank of the Pared River, 50 m, 17°49.967'N, 121°56.042'E.

##### Diagnosis.

A tall, yellowish, very slender diplommatinid with club-shaped apex, dense, low ribs on the last whorl, rounded lower whorls, strongly expanded and reflected peristome that is strongly oblique to the shell axis, and a weak interrupted columellar lamella.

##### Description of the shell

(Figs 1A–H, 2F, H). Shell sinistral, tall, very slender; apex thickened; penultimate whorl wide, body whorl constricted, peristome strongly expanded; whorls 16.5–18; shell colour overall pale yellow or corneous, sometimes seemingly darker due to the desiccated body, subtranslucent; protoconch consists of approximately 1.25–1.5 whorls, finely pitted; first whorls of teleoconch conspicuously narrower than protoconch; teleoconch rather regularly, obliquely ribbed with fine spiral striation, which is most conspicuous on lower whorls; ribs straight on upper whorls but become more wavy on last whorl (especially near suture); upper whorls concave, slowly, rather regularly increasing; constriction deep, situated on penultimate whorl; last whorl conspicuously narrower than preceding whorl; lower whorls rounded; aperture strongly oblique to shell axis, rounded, with a weak columellar lamella visible from standard apertural view; columellar lamella low, interrupted, its inner, separate, blunt thorn-like part situated inside post-constriction bay (widened area just anterior to operculum); no other plicae or lamellae found; peristome overall strongly expanded and reflected; boundary between inner and outer peristome clearly visible due to sharp, usually reddish brown edge of inner peristome; outer peristome mostly responsible for expanded profile of peristome; upper, parietal part of peristome free from penultimate whorl; umbilicus absent.

##### Measurements.

Shell height: 8.7–10.3 mm; shell width: 1.6–1.8 mm; aperture height: 2.2–2.7 mm (n = 6).

**Operculum** (Figs 2A–D). Corneous, flat (not concave); outer surface smooth, without any signs of whorls, but with a very thin matt layer; under matt layer glossy; inner surface overall rather smooth, with a very low arcuate ridge on one side, and a low central nipple, which is also visible from outside (because the operculum is semi-transparent).

**Radula** (Fig. 2E). Radula taenioglossate. Teeth arranged in v-shaped rows, each transverse row with seven teeth (2-1-1-1-2). Rachidian tooth strongly constricted in its middle part, having five cusps (central cusp largest, blunt, other four cusps pointed); inner marginal and two outer marginal teeth have shallower constriction of plates, and are slightly longer and more slender than central tooth; inner marginal teeth with four pointed cusps, third one (counting from the side of rachidian tooth) is largest; outer marginal teeth with four pointed cusps.

##### Etymology.

The shell shape of this new species (wide aperture, very slender upper whorls, and a club-shaped apex) resembles a radio antenna. The specific epithet *antenna* to be used as a noun in apposition.

##### Habitat and distribution.

Living specimens were found on a limestone rock wall. This species is known from the type locality only, which is situated ca. 34 km in a straight line from the type locality of *L. angulata* sp. n.

##### Comparisons.

Most sinistral diplommatinids from the Philippines belong to the genera *Palaina* and *Diancta* Martens, 1867, and have conical, ovoid, or cylindrical shells. However, most *Diplommatina* species from the Philippines are dextral, and the very few sinistral species have much lower spire, and triangular or ovoid shell shape (Zilch 1953). The only similar species in the region is *Luzonocoptis angulata* sp. n., which differs from *L. antenna* sp. n. in the following traits: whorls fewer; lower whorls keeled; aperture less oblique to the shell axis; peristome much less reflected; constriction situated approx. half whorl anteriorly (behind the parietal part of the peristome); ribs more widely-spaced on the neck region; inner, separated part of the columellar lamella blunter.

#### Luzonocoptis angulata

Taxon classificationAnimaliaArchitaenioglossaDiplommatinidae

Páll-Gergely & Hunyadi sp. n.

http://zoobank.org/CB277A41-0983-42BB-BA44-A5515DB37D1B

Figures 1I–P , 2G, I

##### Type material.

Philippines, Luzon, Cagayan Province, 10 km southeast from Gattaran, Barangay Naddungan, environment of the Ar-Aro Cave, rock wall facing east, 90 m, 18°4.477'N, 121°44.128'E, leg. Hunyadi, 08.01.2014. HNHM 99996 (holotype, H = 9.1 mm, D = 2.1 mm), HNHM 99998 (3 paratypes), HA/238 paratypes, PGB/3 paratypes.

##### Type locality.

Philippines, Luzon, Cagayan Province, 10 km southeast from Gattaran, Barangay Naddungan, environment of the Ar-Aro Cave, rock wall facing east, 90 m, 18°4.477'N, 121°44.128'E.

##### Diagnosis.

A tall, yellowish, moderately slender diplommatinid with club-shaped apex, widely spaced, sharp ribs on the last whorl, angled lower whorls, strongly expanded peristome that is slightly oblique to the shell axis, and a weak interrupted columellar lamella.

##### Description of the shell

(Figs 1I–P, 2G, I). Shell sinistral, tall, very slender; apex thickened; penultimate whorl wide, body whorl constricted, peristome strongly expanded; whorls 14–15; shell colour overall pale yellow or corneous to light reddish, sometimes seemingly darker due to the desiccated body, subtranslucent; protoconch consists of approximately 1.25–1.5 whorls, finely pitted; first whorls of teleoconch conspicuously narrower than teleoconch; first whorls of teleoconch with low, irregular growth wrinkles, which gradually change to a scarcely, regularly ribbed surface; some weak spiral striation visible between ribs; ribs are strongest on last whorl, where they are sometimes lamella-like; upper whorls slightly concave or not concave, slowly, rather regularly increasing; constriction very deep, situated between penultimate and last whorl (just behind parietal part of peristome); deep constriction results in formation of a conspicuous post-constriction bay (widened area just anterior to operculum); last whorl conspicuously narrower than preceding whorl; lower 3–4 whorls slightly keeled at their middle; aperture moderately oblique to shell axis, rounded, with a weak columellar lamella visible from standard apertural view; columellar lamella low, interrupted; its inner, separate, elongated tubercle-like part situated inside post-constriction bay; no other plicae or lamellae found; peristome overall strongly expanded but varies from not reflected to slightly reflected; boundary between inner and outer peristome clearly visible due to sharp edge of inner peristome; outer peristome mostly responsible for expanded profile of peristome; upper, parietal part of peristome is attached to penultimate whorl, although expanded part extends above penultimate whorl; umbilicus absent.

##### Measurements.

Shell height: 7.8–9.3 mm; shell width: 1.8–2.1 mm; aperture height: 2.2–2.6 mm (n = 5).

**Operculum.** Unknown.

##### Etymology.

The specific epithet *angulata* (Latin: angled) refers to the keeled lower whorls, which distinguishes this species from *L. antenna* sp. n.

##### Habitat and distribution.

Empty shells were found at the base of a limestone rock wall. This species is known from the type locality only, which is situated ca. 34 km in a straight line from the type locality of *L. antenna* sp. n.

##### Comparisons.

See under *Luzonocoptis antenna* sp. n.

## Discussion

Although molecular phylogenetic studies involving numerous species were recently published (Webster et al. 2012, Liew et al. 2014), molecular information is still lacking on some important members of the family, such as the type species of *Diplommatina* from the southwestern Himalaya and the type species of several other genus-group taxa, mainly from oceanic islands. As a result, we still largely have to rely on morphology-based taxonomy of diplommatinid species. Placing the two new species described herein into any previously established genera would not be possible without strongly affecting the existing generic definitions (Páll-Gergely 2017). Therefore, from a typological perspective, a new genus, *Luzonocoptis* gen. n. must be introduced for them.

The two *Luzonocoptis* gen. n. species are similar to species of the Palawan endemic genus *Hungerfordia* (type species: *Hungerfordia pelewensis* Beddome, 1889) in the rock-dwelling lifestyle, the sinistral coiling direction, the absence of parietal and palatal plicae or lamellae, and the low arcuate ridge on the inner surface of the operculum (Yamazaki et al. 2013, 2015a, 2015b). Although the slender shell having numerous (14–18) whorls is unique to the two new *Luzonocoptis* gen. n. species, it is insufficient alone for genus-level distinction from the conchologically diverse *Hungerfordia*. However, the interrupted columellar lamella, and the rachidian tooth having five well-developed cusps justifies the distinction of the two new species from *Hungerfordia* species on the genus level.

## Supplementary Material

XML Treatment for Luzonocoptis

XML Treatment for Luzonocoptis antenna

XML Treatment for Luzonocoptis angulata
